# Practical Chemistry for Students and Physicians—Chapter Sixth

**Published:** 1883-12

**Authors:** 


### Editorial.

### PRACTICAL CHEMISTRY FOR STUDENTS AND PHYSICIANS—CHAPTER SIXTH.

(Continued from November Register, Vol. III, Page 54.)

*Classification of the Non Metals.*—We will now consider as many of the non-metalic elements as are of practical importance. These are as follows in the order of their classes and groups:

CLASS I.

Group I, Hydrogen H.

Group II, Oxygen O.

CLASS II.

Group I, Clorine Cl. Bromine Br. Iodine I. Fluorine F.

Group II, Sulphur S. Selerium Se.

Group III, Nitrogen N. Phosphorus P. Arsenic A. Antimony Sb.

Group IV, Boron B.

Group V, Carbon C. Silicon Si.

They are all found in two classes and seven groups, and the basis of this classification is similarity of chemical characteristics, excepting hydrogen and oxygen, which occupy the first class solely in virtue of their unequaled typical and practical value in chemistry and physics.

Hydrogen is, now, the great standard of atomic weight, molecular weight, molecular volume, and of density and quantivalence. It possesses the highest capacity of all bodies for heat, and combining with oxygen, yields the highest known intensity per gramme, of sensible heat; while oxygen by its universal prevalence, and unsur. passed chemical activity, is scarcely less important than hydrogen.

*The Basis of the Classification.*—Two of the non-metalic elements, therefore, constitute the first class, because of their superior importance, and the remainder in great part, the second class, in virtue of the important fact that their oxides in union with water, form acids only, never a base nor a salt with an oxacid. There are several apparent metalic elements named in class second of this arrangement of the elemental bodies, but they are not, now, so regarded, any more than arsenicum and stibium.

The acids of these non-metals form oxysalts with the metalic elements; as $K_2 HAO_3$ or $No._3 AsO_4$, but not one of them has been known to unite with an acid having oxygen. There are no such compounds as $AsPO_4$ arsenicum phosphate, or $P(No._3)_3$, nitrate of phosphorus.

The remaining two groups, not here named of this class, are all save two or three elements, exceedingly rare.

The members of the first class hydrogen and oxygen having been already noticed sufficiently for present purposes, it is in order to consider the characteristics, properties and uses of those of the second, beginning with bromine, since chlorine, the first element in the first group, was included, as a gas, in the notice given of hydrogen, oxygen and nitrogen.

*General View of the Halogen Elements. First Group of Class II.*—These are fluorine, chlorine, bromine, and iodine, written in the order of their chemical activity. So closely are they related in their chemical character and behavior, that they form the most remarkable family group in chemistry. This is indicated, so far as nomenclature can affect it, by the common terminal ene appended to the name of each. The family name of halogen was given them to commemorate their relation to the sea, *als* or *hals* in the Greek meaning the sea and *gens* kind or family. They are sometimes called *salt radicals*, because by direct union with the radicals, as in NaCl, sodium chloride, they form the *haloid salts*.

They are, each, monotomic in their quantivalence, combining with a single atom of hydrogen to form an acid —a hydracid—having a volume equal to the sum of the volumes of its constituents.

It is not the least curious of this group's peculiarities, that their atomic weights should increase inversely as their chemical energies: fluorine, 19; chlorine, 35.5, bromine, 80, and iodine 127. Hence it may be said, too, that their densities increase inversely with their chemical activity since the same numbers express their respective relative weights in vapor, hydrogen being 1.

There is no other natural group in chemistry, whose chemical and physical properties, present so striking a gradation and so many typical features. Every physical condition finds its representative here; is any element or compound a gas? so is chlorine; is any a solid? so is iodine; is any a liquid? bromine is such; is the compound radical ammonium unisolated and hypothetical? so is fluorine.

The gradation of their chemical properties, is no less remarkable. Fluorine stands at the head of the group if force of atomic attraction, and molecular stability are the measure of chemical activity. This energy is so great that it has thwarted all attempts to isolate it satisfactorily. Chlorine ranks second in its combining force; bromine third and iodine last.

Physically, again, chlorine is greenish-yellow, bromine red, and iodine brownish-black with some of the luster of a metal. Bromine boils at 145°F, and iodine at 347°F.

These elements play, also, a graduated part with oxygen; fluorine will have none of it, there is no oxide of fluorine; chlorine attracts it less than bromine; hydrogen chlorate, $HClO_3$ is much less stable than hydrogen bromate, $HBrO_3$, whilst bromine attracts it less than iodine. It is asserted that iodine is capable of uniting directly with the oxygen allotrope, ozone, to form $HIO_3$, iodic acid.

*Fluorine, Symbol F. Atomic Weight* 19. *Quantivalence* 1. *Molecular Weight* 38(?) *Density* 19(?) *Molecular Volume* 2(?). —Every constituent of this element, it will be observed, except its symbol and atomic weight, is marked hypothetical for the simple reason, that it has never been isolated and weighed and measured with hydrogen. Fluorine is chiefly known in its combination with lime in the mineral fluor or Derbyshire spar, fluorite, found abundantly in the county of Derbyshire, England; hence its usual name. It is sometimes called fluor spar or fluorite, from the Latin fluo, I flow, because it is frequently used as a flux in the smelting of copper and other metals.

The cubical crystals of fluorite, with their rich variety of yellow, blue, rose-red, pink, purple or orange colors, often phosphorescent, are very beautiful, and in Derbyshire are frequently made into vases, candlesticks and other ornaments. There is no fluor-spar in Georgia; it is abundant in New England and New York.

*Compounds and Uses.*—These require but a brief notice. In union with hydrogen, fluorine forms, HF, hydrogen fluoride, the so-called hydrofluoric acid.

*Preparation.*—It is usually prepared from Derbyshire spar, calcium fluoride, by the action of sulphuric acid: $CaF_2 \times H_2SO_4 = CaSO_4 \times 2HF$. The process necessitates a vessel of lead or platinum, glass being readily attacked by the resulting gas, HF. As chlorine loves hydrogen, fluorine is attracted by silicon, in union with oxygen the chief constituent of glass; no sooner than the gaseous HF is generated than it begins to corrode any glass that may be present in search of silion. The following is its reaction with silica: $4HFxSiO_2 = 2H_2OxSiF_4$.

The principal use of fluorine,therefore, is in etching on glass, and for this purpose there is no other agent known that can take its place. All the beautiful tracery and figuring seen upon glass at this day, and upon many gems of the silicates, are the effects of HF.

*The Process.*—This is quite simple. Bend a piece of sheet lead into the form of a basin; place in this a few grains of powdered fluor-spar, and wet it to a paste with sulphuric acid. A gentle heat applied to the basin will cause the gas to be evolved at once.

The glass is thus prepared: cover the side to be etched with wax, trace the letters or figures with a pointed instrument and lay the glass, wax side down, upon the basin. The rising vapor reaches the glass through the tracery in the wax and in a very few minutes does its work. No engraver with an instrument of steel, could cut the lines so smoothly and neatly.

The wax is now cleansed away by a little warm oil of turpentine.

This art is very valuable to the chemist for imparting permanent labels to his reagent bottles.

Like HCl gaseous HF is very soluble in water and imparts to the solution all of its chemical properties. If it be applied to glass in this form, the effect is smoother lines or surfaces; when the gas is evolved against a surface, it is roughened.

*HF an Irritant Poison.*—When strong it powerfully corrodes the skin, producing ulcers both painful and difficult to heal; and if inhaled as a gas, is more deliterious to the air passages than either chlorine or HCl.

Here carefulness in manipulation is the proper antidote.

